# F191. THE GENETICS OF DRUG-RELATED MOVEMENT DISORDERS AN UMBRELLA REVIEW OF META-ANALYSES

**DOI:** 10.1093/schbul/sby017.722

**Published:** 2018-04-01

**Authors:** Nadine van der Burg, P Roberto Bakker, Jim Van Os, Peter Van Harten, Asmar Al Hadithy

**Affiliations:** 1 GGZ Centraal; 2 Maastricht University; 3 Maastricht University Medical Centre; 4 Parnassia Group

## Abstract

**Background:**

Treatment with antipsychotics can provoke drug-related movement disorders (DRMD) (also known as extrapyramidal symptoms (EPS)), i.e. tardive dyskinesia (TD), parkinsonism, akathisia and tardive dystonia. DRMD remain a cause for concern in the treatment of patients with psychotic disorders, especially because the DRMD can become irreversible (Correll and Schenk, 2008). There are lower percentages in younger patients (32%) (Mentzel 2017), but the prevalence is substantial in chronic patients (68%) (Bakker 2011), with around a quarter of chronic patients showing two different types of DRMD (Bakker 2011). DRMD can cause severe impairment in quality of life (Fujimaki 2012. In addition, a meta-analysis (Ballesteros 2000) and two recent studies showed a higher mortality in patients with tardive dyskinesia (Chong 2009, Dean and Thuras 2009). It is therefore important to find ways of preventing DRMD. Pharmacogenetic studies may identify genetic risk factors, which underlie individual vulnerability for DRMD in response to antipsychotics (Reynolds 2007; Ohmori 2003; Lerer 2002), in theory paving the way for individually tailored medication prescriptions (Lerer and Segman 2006). To date, many different papers have been written on the subject and they have presented inconsistent results.

The aim of this umbrella review is to provide clinicians and patients with evidence-based information regarding the genes that are thought to be associated with DRMD and to use this umbrella review on the genetics of DRMD as a basis for recommendations for future prevention programs and research.

**Methods:**

To identify all relevant meta-analyses a Medline, Embase, and Psychinfo literature search was performed. Titles and abstract were screened using predetermined criteria by two independent authors. The methodological quality of included meta-analyses was assessed by two overview authors using ‘assessment of multiple systematic reviews’ (AMSTAR) critical appraisal checklist. Reference lists of included papers and those of reviews were cross-checked and no new publications were found.

**Results:**

The search yielded 14 meta-analysis studies and consensus was obtained. The DRD3, DRD2, CYP2D6, 5-HT2A, COMT and MnSOD genes all contain variants that increase the odds ratio of TD. However meta-analyses showed diminishing significance over time and meta-analyses on the same subject were difficult to compare due to differences in patient population and methods used.

**Discussion:**

For now it appears that TD is a complex disease with multiple genes that are involved in its phenotype and more studies (eg. Genome wide associatio studies), on a larger scale, are required to develop a genetic test kit to predict the chance of TD. To achieve this multiple research groups need to work together, a DRMD genetic database needs to be in place to overcome publication bias and results need to be stratified by patient characteristics.

The result could be test that undoubtedly will be of great clinical value in treating patients and by prospectively preventing debilitating DRMD.

